# A Spatially Distributed Perturbation Strategy with Smoothed Gradient Sign Method for Adversarial Analysis of Image Classification Systems

**DOI:** 10.3390/e28020193

**Published:** 2026-02-09

**Authors:** Yanwei Xu, Jun Li, Dajun Chang, Yuanfang Dong

**Affiliations:** 1 School of Management Science and Information Engineering, Jilin University of Finance and Economics, Changchun 130117, China; xuyanwei@s.jlufe.edu.cn; 2 School of Railway Locomotives and Rolling Stock, Jilin Tiedao University, Jilin 132299, China; changdajun@mails.cust.edu.cn; 3 School of Economics and Management, Changchun University of Science and Technology, Changchun 130022, China; dyf@cust.edu.cn

**Keywords:** adversarial examples, adversarial attacks, deep learning, computer vision, complex systems, systems assurance

## Abstract

As deep learning models are increasingly embedded as critical components within complex socio-technical systems, understanding and evaluating their systemic robustness against adversarial perturbations has become a fundamental concern for system safety and reliability. Deep neural networks (DNNs) are highly effective in visual recognition tasks but remain vulnerable to adversarial perturbations, which can compromise their reliability in safety-critical applications. Existing attack methods often distribute perturbations uniformly across the input, ignoring the spatial heterogeneity of model sensitivity. In this work, we propose the Spatially Distributed Perturbation Strategy with Smoothed Gradient Sign Method (SD-SGSM), a adversarial attack framework that exploits decision-dependent regions to maximize attack effectiveness while minimizing perceptual distortion. SD-SGSM integrates three key components: (i) decision-dependent domain identification to localize critical features using a deterministic zero-out operator; (ii) spatially adaptive perturbation allocation to concentrate attack energy on sensitive regions while constraining background disturbance; and (iii) gradient smoothing via a hyperbolic tangent transformation to enable fine-grained and continuous perturbation updates. Extensive experiments on CIFAR-10 demonstrate that SD-SGSM achieves near-perfect attack success rates (ASR 99.9%) while substantially reducing $\ell_{2}$ distortion and preserving high structural similarity (SSIM 0.947), outperforming both single-step and momentum-based iterative attacks. Ablation studies further confirm that spatial distribution and gradient smoothing act as complementary mechanisms, jointly enhancing attack potency and visual fidelity. These findings underscore the importance of spatially aware, decision-dependent adversarial strategies for system-level robustness assessment and the secure design of AI-enabled systems.

## 1. Introduction

With the rapid advancement of computer technology and artificial intelligence, deep learning has become a core enabling technique in modern intelligent systems, achieving remarkable performance in a wide range of tasks such as image classification and visual recognition. High-accuracy deep neural networks (DNNs) have been increasingly deployed in real-world applications, including autonomous driving, intelligent surveillance, and biometric authentication, where reliable perception and decision-making are of critical importance. However, despite their impressive predictive capability, DNNs have been shown to be highly vulnerable to carefully crafted adversarial perturbations [1,2]. Such perturbations are often imperceptible to human observers yet can significantly distort model predictions, thereby undermining the robustness and trustworthiness of intelligent systems. This vulnerability raises serious concerns for safety-critical and security-sensitive scenarios, where erroneous decisions may lead to substantial social, economic, or ethical consequences [3,4].

From a system security and decision-support perspective, the vulnerability of deep neural networks further exposes fundamental limitations in existing adversarial analysis and evaluation methodologies. Traditional adversarial attack methods [5,6,7], such as FGSM and PGD, typically assume a uniform perturbation cost across the entire input space. This assumption implicitly treats all input dimensions as equally influential to the model’s decision process, thereby neglecting the fact that DNNs often exhibit pronounced spatially heterogeneous sensitivity. In practice, only a subset of input regions may play a dominant role in determining the final prediction, while perturbations applied to other regions contribute marginally to decision changes.

Inspired by the occlusion sensitivity technique introduced by Zeiler and Fergus (ECCV 2014) [8], which systematically evaluates the impact of masking localized regions on model predictions, we adopt a similar intuition to probe spatially heterogeneous decision dependencies in adversarial settings. Specifically, we reformulate this idea through a deterministic Local Zero-Out Operator, which selectively suppresses localized regions to assess their influence on model confidence. The spatial regions identified through this process are referred to as decision-critical domains.

The decision-critical domains, referring to localized regions that disproportionately affect model confidence and decision outcomes. Perturbations concentrated within these domains can significantly degrade classification reliability while inducing minimal global distortion, which suggest that spatially aware perturbation strategies are essential for accurately evaluating and understanding model robustness.

Motivated by these insights, we propose a Spatially Distributed Perturbation Strategy with Smoothed Gradient Sign Method (SD-SGSM), a unified adversarial framework designed to align perturbation generation with the intrinsic decision structure of deep neural networks. Specifically, SD-SGSM integrates three key components: (1) decision-dependent domain identification, which localizes model-critical regions through a deterministic zero-out operator; (2) spatially adaptive perturbation allocation, which concentrates attack energy on high-sensitivity domains while suppressing unnecessary background disturbance; and (3) gradient smoothing via a hyperbolic tangent transformation, enabling fine-grained and continuous perturbation updates. Furthermore, we extend the proposed strategy into a multi-step iterative framework, allowing adversarial examples to be progressively refined under strict per-pixel cost constraints. This design effectively balances attack efficacy with perceptual imperceptibility, addressing both security evaluation and decision reliability concerns in intelligent systems.

Experiments conducted on benchmark datasets demonstrate that SD-SGSM outperforms conventional single-step and multi-step adversarial attack methods. In particular, the proposed approach achieves higher Attack Success Rates (ASR) while simultaneously reducing global $\ell_{2}$ and $\ell_{\infty }$ norms. These empirical results validate the practical effectiveness of spatially aware and decision-dependent perturbation strategies and underscore their importance in advancing robustness evaluation, risk assessment, and trustworthy decision support for deep learning-based intelligent systems.

## 2. Related Works

Adversarial attacks are systematically classified into distinct categories based on various criteria. A primary distinction is established based on the adversary’s knowledge of the model’s internal architecture, resulting in two principal paradigms: white-box attacks and black-box attacks [9]. A white-box attack is predicated on the assumption that the adversary possesses comprehensive knowledge of the target model’s internal structure and gradient information to synthesize adversarial samples. Extensive research has demonstrated that white-box attacks can craft adversarial examples with a high success rate [10]. In contrast, a black-box attack is conducted without access to the internal structure or gradient derivatives of the targeted model. However, adversarial examples generated in white-box settings often exhibit limited transferability when applied to black-box models protected by defensive mechanisms [10]. Furthermore, attacks can be categorized by the intended outcome: targeted attacks are engineered to force the model to misclassify input data into a specific, predetermined class under defined constraints, whereas non-targeted attacks aim solely to induce misclassification without a specific target label constraint.

The continuous evolution of adversarial defense mechanisms has necessitated the development of increasingly sophisticated attack algorithms. Ensuring the robustness and security of deep learning models requires the deployment of more potent adversarial attack methodologies. Although deep neural networks (DNNs) have achieved remarkable performance, distinct vulnerabilities have been uncovered in multiple state-of-the-art architectures. For instance, Convolutional Neural Networks (CNNs), despite being trained meticulously for image classification, have been shown to perform disastrously when subjected to adversarial attacks [11,12]. Similar fragility has been observed in other domains: neural retrieval models are brittle when faced with distribution shifts or malicious attacks [13], and plant disease classification models remain susceptible to robustness issues [14]. Adversaries exploit these intrinsic blind spots to generate adversarial examples capable of misleading machine learning models through imperceptible perturbations to the input data distribution [10].

### 2.1. White-Box Attacks

In the realm of gradient-based white-box attacks, the Fast Gradient Sign Method (FGSM), proposed by Goodfellow et al. [15], serves as a foundational baseline. Predicated on the linearization of the loss function, FGSM is capable of rapidly generating adversarial examples. However, this method requires the manual selection of a perturbation coefficient, and its linearity assumption along with a fixed perturbation magnitude often results in suboptimal adversarial examples [16]. To mitigate these limitations, the Iterative Fast Gradient Sign Method (I-FGSM), proposed by Kurakin et al. [17], enhances FGSM by applying perturbations iteratively. While this approach typically yields smaller perturbations and stronger white-box attack capabilities compared to FGSM, it incurs a higher computational overhead. To further enhance attack success rates (ASR) in white-box scenarios, recent studies have introduced advanced optimization strategies. The Trans-IFFT-FGSM, presented by Naseem [18], incorporates multiple modules to retain input noise information, improving ASR at the cost of increased algorithmic complexity. In domain-specific applications, the GP-MI-FGSM utilizes gamma correction and image pyramids to improve success rates in plant disease classification [14], while Genetic Algorithms have been combined with FGSM to iteratively optimize the epsilon value for robust adversarial example generation in remote sensing [16].

### 2.2. Black-Box Attacks

In black-box settings where gradients are unavailable, attacks often rely on the transferability of adversarial examples generated from substitute models. To address the transferability issue, the AB-FGSM integrates the AdaBelief optimizer into the iterative framework to identify transferable adversarial points across different optimization surfaces [10]. Similarly, the SAMI-FGSM employs stochastic gradient accumulation to stabilize the update direction, thereby achieving higher transferability in black-box settings [19]. These methods demonstrate that enhancing the generalization of gradients on source models can effectively compromise unknown target models.

### 2.3. Adversarial Defenses

In the study of adversarial attacks, the development of attack algorithms serves a critical purpose: to expose model vulnerabilities and, through the study of adversarial defense algorithms, to enhance model robustness and ultimately improve system security. Adversarial defense refers to the techniques designed to protect deep neural networks from maliciously crafted inputs that aim to deceive the model [20]. Its primary goal is to maintain the model’s robustness and reliability in the presence of such attacks [21]. The most fundamental and widely adopted approach is adversarial training [22], which enhances robustness by incorporating adversarial examples during training. This method reduces loss curvature and narrows the robustness gap between training and test data [21]. Variations and extensions include regularization-based techniques such as Local Linear Regularization (LLR) [23] and Input Gradient Regularization (IGR) [24]. Other notable strategies include Topology-Aligned Adversarial Training (TAAT) [25], Feature-Decoupled Networks (FDNet), Attention-Guided Reconstruction Loss (AIAF-Defense) [26], as well as feature denoising [27], domain adaptation [28], and ensemble defenses [21]. In short, these methods form a multi-faceted defense landscape aimed at securing deep learning systems against evolving adversarial threats.

## 3. Method

### 3.1. Overview

Standard adversarial attacks (e.g., FGSM, PGD) typically assume a uniform perturbation cost across the entire image lattice. However, deep neural networks exhibit spatially non-uniform sensitivity—certain “evidence regions” contribute disproportionately to the classification decision.

**Figure 1:**
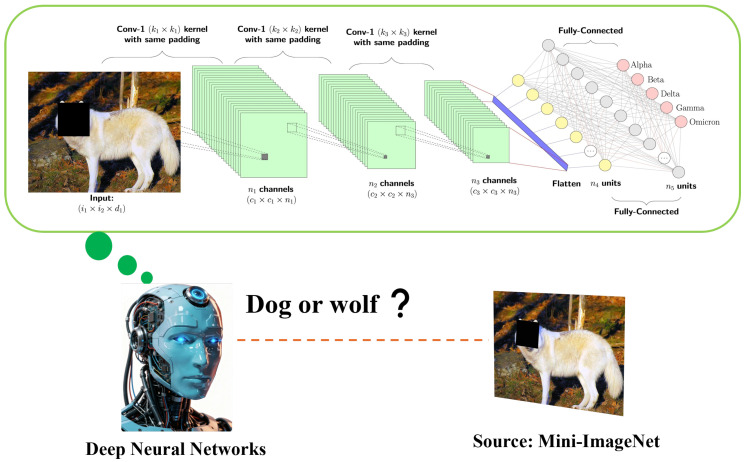
Although DNNs are robust to random occlusions via data augmentation, blocking core regions (e.g., the face) significantly lowers confidence, making the model unsure if the image is a “dog” or a “wolf”. We exploit this by searching for regions that cause such confidence drops to guide our perturbation.

Although deep neural networks utilize data augmentation techniques to bolster robustness against general occlusions, their prediction confidence deteriorates significantly if the core semantic region of the image is obstructed. As illustrated in Figure 1, when the facial features of a dog are partially masked, the neural network becomes unable to distinguish whether the subject is a “dog” or a “wolf”. This phenomenon reveals a critical insight: while the model is robust to peripheral noise, the loss of key structural evidence leads to ambiguous classification.

Motivated by this observation, we propose a Spatially Distributed Gradient Smoothing (SD-SGSM) framework. We specifically leverage the occlusion sensitivity demonstrated in Figure 1 to guide our attack generation through three integrated components:

**Decision-Dependent Domain Identification.** Driven by the “dog or wolf” ambiguity caused by masking, we employ a Local Zero-Out Operator. This mechanism acts as a deterministic spatial dropout that systematically excises local patches. By identifying the locations where removal causes the lowest confidence (simulating the masked dog face), we precisely localize the model’s decision-dependent domains.

**Spatially Distributed Perturbation Strategy.** Having located these critical regions, we abandon the uniform cost approach. Instead, we concentrate the attack energy by assigning a larger perturbation cost ($\epsilon_{\mathrm{strong}}$) exclusively to these high-sensitivity domains, while restricting the remaining non-sensitive background to a lower cost ($\epsilon_{\mathrm{weak}}$).

**Smoothed Gradient Sign Method (SGSM).** To optimize these spatially varying perturbations effectively, we replace the standard sign operator with a hyperbolic tangent function to mitigate gradient quantization errors.

### 3.2. Decision-Dependent Domain Identification

Given an input image $x\in R^{H\times W\times C}$ and a pre-trained classifier $f:R^{H\times W\times C}\to R^{K}$, our primary objective is to identify spatial regions—termed decision-dependent domains—where the removal of features induces a significant shift in the model’s output distribution.

**Local Zero-Out Operator.** To analyze local feature importance without introducing exogenous noise patterns, we select Ω as a fixed-size square window occupying a relatively small fraction of the original image. To avoid a significant degradation in attack efficiency, the operator is applied in a sliding-window manner with a stride equal to the patch size, ensuring non-overlapping patches while maintaining computational efficiency. We employ a masking strategy. Let Ω⊆{1,…,H}×{1,…,W} denote a contiguous spatial patch, we define the Local Zero-Out Operator $Z_{\Omega }:R^{H\times W\times C}\to R^{H\times W\times C}$ as

(1) $${\left(Z_{\Omega }\left(x\right)\right)}_{i,j,c}=\left\{\begin{array}{cc} 0, & (i,j)\in \Omega ,\\ x_{i,j,c}, & \mathrm{otherwise}. \end{array}\right.$$

This operator functions as a deterministic, spatially localized analogue of dropout, excising entire structural evidence units.

**Decision Deviation Metric.** We quantify the contribution of region Ω by measuring the divergence between the logits of the original and the occluded images. In this work, *f* refers to a pre-trained deep neural network–based image classifier commonly used in visual recognition tasks. We define the decision deviation metric $\Delta_{\Omega }$ using the $\ell_{2}$ distance as follows:

(2) $$\Delta_{\Omega }={∥f\left(x\right)-f(Z_{\Omega }\left(x\right))∥}_{2}.$$

The magnitude of $\Delta_{\Omega }$ serves as a proxy for the model’s reliance on the visual evidence contained within Ω. A region is designated as decision-dependent if its removal causes a deviation exceeding a stability parameter τ (i.e., $\Delta_{\Omega }>\tau$).

**Scanning-Based Approximation.** Identifying the optimal region $\Omega^{★}=\arg\max_{\Omega }\Delta_{\Omega }$ via exhaustive search is computationally prohibitive. To ensure efficiency, we approximate the search space P using a sliding window approach with non-overlapping patches of size m×m. This yields a discretized saliency map $\left\{\Delta_{\Omega_{k}}|\Omega_{k}\in P\right\}$, identifying the principal decision-dependent domain $\Omega^{★}$ in a forward-pass efficient manner.

### 3.3. Spatially Distributed Perturbation Strategy

In norm-bounded adversarial attack settings, the perturbation magnitude is typically constrained by an $\ell_{\infty }$ cost to ensure imperceptibility, which can be expressed as

(3) $${∥\delta ∥}_{\infty }\leq \epsilon .$$

As shown in Equation (3), this global constraint enforces an inherent trade-off: a small ϵ preserves visual quality but limits attack success in robust regions, whereas a large ϵ improves attack effectiveness at the cost of perceptual degradation in smooth background areas.

To address this issue, we propose a dual-cost perturbation strategy. Based on the identified sensitive domain $\Omega^{★}$, the image lattice is partitioned into a sensitive region set *R* (corresponding to $\Omega^{★}$ and its high-response neighbors) and a non-sensitive background set $\bar{R}$. Differential perturbation costs are then assigned to these two regions:

(4) $$x_{\mathrm{adv}}=x+\delta ,\;\delta_{i,j}\in \left\{\begin{array}{cc} {}[-\epsilon_{\mathrm{strong}},\epsilon_{\mathrm{strong}}], & (i,j)\in R,\\ {}[-\epsilon_{\mathrm{weak}},\epsilon_{\mathrm{weak}}], & (i,j)\notin R, \end{array}\right.$$

where $\epsilon_{\mathrm{strong}}>\epsilon_{\mathrm{weak}}$.

For notational convenience, we define a pixel-wise perturbation cost

(5) $$\epsilon \left(i,j\right)=\left\{\begin{array}{cc} \epsilon_{\mathrm{strong}}, & (i,j)\in R,\\ \epsilon_{\mathrm{weak}}, & (i,j)\notin R. \end{array}\right.$$

This strategy concentrates the attack on the decision-dependent domain to maximize misclassification, while maintaining a lower perturbation profile in the background to preserve overall perceptual fidelity (measured by $\ell_{p}$ norms).

### 3.4. Smoothed Gradient Sign Method (SGSM)

The standard FGSM relies on the sign(·) operation to normalize gradients. While efficient, the sign function is non-differentiable at zero and induces “gradient quantization”, where fine-grained gradient information is lost to binary directions −1 or +1.

**Hyperbolic Tangent Smoothing.** We propose replacing the sign projection with a scaled hyperbolic tangent function, which introduces smoothness and differentiability into the perturbation update:

(6) sign(g)⟶tanh(ω·g),

where $g=\nabla_{x}J\left(\theta ,x,y\right)$ is the gradient of the loss function, and ω is a scaling factor controlling the steepness (or saturation) of the activation.

**Update Rule.** Incorporating the spatially distributed perturbation strategy defined in Section 3.3, the adversarial example is updated as

(7) $$x_{\mathrm{adv}}\left(i,j\right)={\mathrm{Clip}}_{\left[0,1\right]}\left(x\left(i,j\right)+{\mathrm{Clip}}_{\left[-\epsilon (i,j),\;\epsilon (i,j)\right]}\left(\alpha \;\tanh(\omega \;\nabla_{x(i,j)}J\left(\theta ,x,y\right))\right)\right),$$

where α denotes the step size, and ϵ(i,j) is the pixel-wise perturbation cost defined in Equation (5).

**Figure 2:**
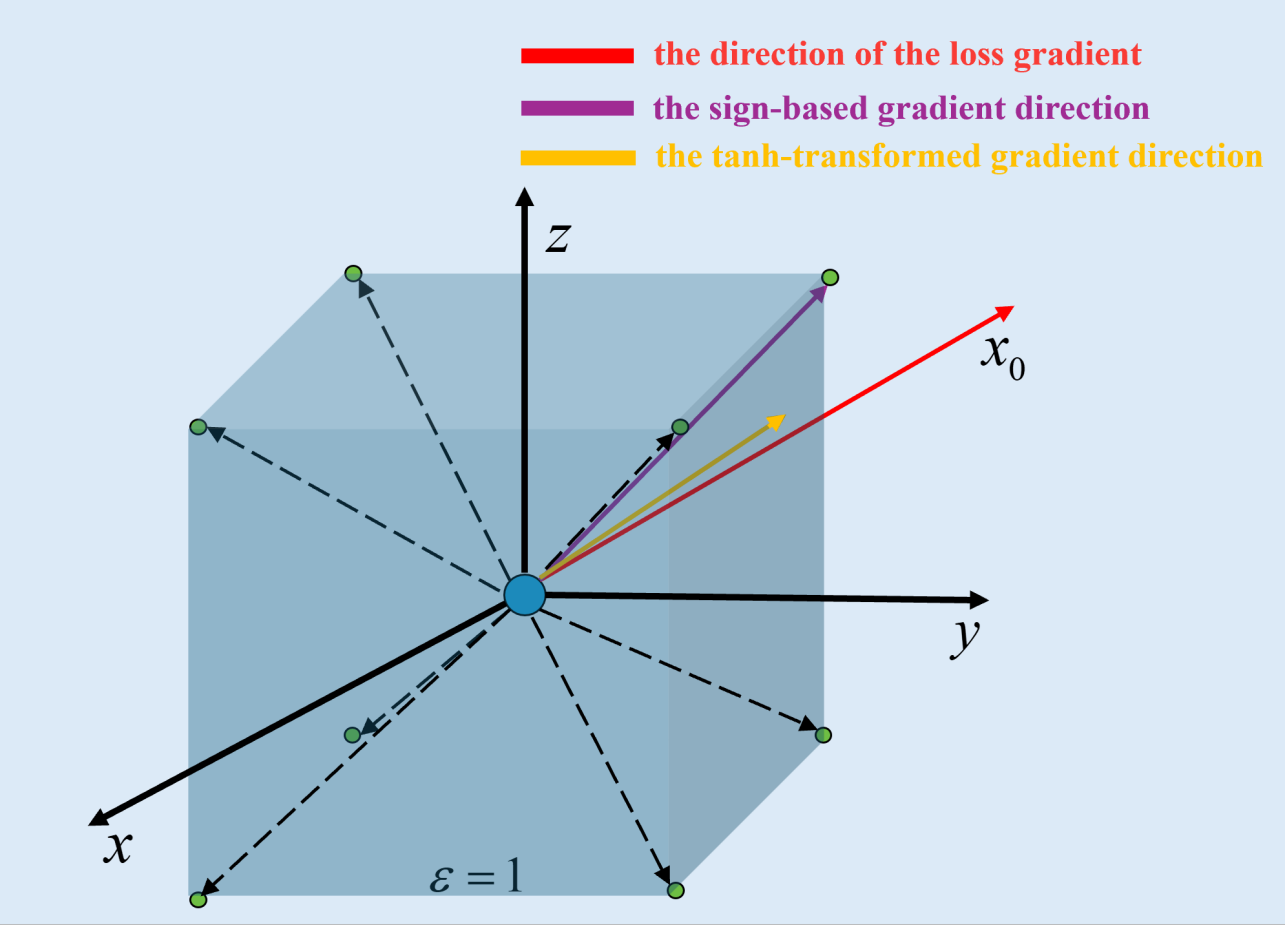
Geometric comparison of gradient directions. The dashed arrows pointing to the cube vertices represent the discrete gradient directions limited by the sign(·) operation in FGSM. The solid red arrow represents the continuous gradient direction enabled by the tanh smoothing, thereby expanding the range of viable gradient directions within the hypercube.

**Geometric Interpretation.** Figure 2 illustrates the geometric advantage of our approach compared to standard FGSM. In the traditional FGSM framework, the gradient direction is constrained to the diagonals of the high-dimensional hypercube (represented by the dashed arrows pointing strictly to the vertices). This limits the perturbation to a discrete set of directions.

In contrast, by introducing the tanh function and additional scaling parameters, our method expands the feasible gradient selection from the vertices to the entire interior volume of the hypercube. As shown by the solid red arrow, the direction is no longer forced to snap to a corner but can point continuously in any optimal direction. This flexibility results in more fine-grained perturbations, effectively reducing the magnitude of noise required to fool the model while ensuring a smoother and more stable optimization trajectory.

To illustrate the advantage of the proposed smoothing in Equation (6), we consider a simple toy example. Suppose a 1-dimensional gradient *g* takes values [−0.2,−0.05,0,0.05,0.2]. Using the standard FGSM sign operation, we have

sign(g)=[−1,−1,0,1,1],

which collapses small and large gradients into the same perturbation direction and ignores relative magnitudes. In contrast, applying the proposed tanh smoothing with a scaling factor ω=5 gives

tanh(ω·g)≈[−0.762,−0.245,0,0.245,0.762],

which preserves the relative strength of each gradient while maintaining a smooth transition around zero. The tanh-based operator retains more informative gradient signals, leading to more precise and stable local perturbations.

### 3.5. Theoretical Analysis: $L_{2}$ Norm Reduction

We next examine the geometric effect of introducing a smooth perturbation direction based on tanh(ωg). Our goal is to quantify how this modification reshapes the perturbation energy while preserving the first-order influence on the loss function. As shown below, under mild assumptions on the gradient scale, the resulting perturbation necessarily exhibits a smaller $L_{2}$ norm than its FGSM counterpart.

Let $g=\left(g_{1},\ldots ,g_{n}\right)\in R^{n}$ denote the gradient with respect to the input *x*. The FGSM update under an $\ell_{\infty }$ constraint ε takes the form

(8) $$\delta_{\mathrm{FGSM}}=\varepsilon \cdot \mathrm{sign}\left(g\right),\;{∥\delta_{\mathrm{FGSM}}∥}_{2}=\varepsilon \sqrt{n},$$

where we assume $g_{i}\neq 0$ to ensure the sign is well-defined.

To introduce a smoother and gradient-sensitive direction, we consider the element-wise mapping s=tanh(ωg) and construct a perturbation

(9) $$\delta_{\tanh}=\alpha \tanh\left(\omega g\right),$$

We define the proposed tanh-based perturbation (element-wise) as s=tanh(ωg). To ensure a fair comparison, this direction is scaled by a factor α>0 such that it achieves the same first-order gain (contribution to the loss change) as FGSM:

(10) $$\langle g,\delta_{\tanh}\rangle =\langle g,\delta_{\mathrm{FGSM}}\rangle .$$

Substituting Equations (8) and (9) into the condition in Equation (10) yields

(11) $$\alpha \langle g,\tanh\left(\omega g\right)\rangle =\varepsilon \sum_{i=1}^{n}|g_{i}{|=\varepsilon ∥g∥}_{1}.$$

Before formally stating the result, we note that when ω is small, the tanh function operates approximately linearly. In this regime, the perturbation direction remains aligned with the gradient, yet the element-wise scaling inherently reduces the overall $L_{2}$ energy compared to the original FGSM update. This observation motivates the following proposition:

**Proposition 1**  (Strict Inequality in the Linear Regime)**.** *When ω>0 is sufficiently small for $\tanh(\omega g_{i})$ to operate in its linear regime, i.e., $\tanh\left(\omega g_{i}\right)\approx \omega g_{i}$ for all i, the Tanh-based perturbation necessarily satisfies*

(12) $$∥\delta_{\tanh}{∥}_{2}^{2}\leq \varepsilon^{2}n={∥\delta_{\mathrm{FGSM}}∥}_{2}^{2}.$$

*The inequality is strict except in the degenerate case where all gradient magnitudes $\left\{|g_{i}|\right\}$ are identical.*

A detailed derivation is presented in Appendix A.2.

The smoothed perturbation operator $\delta_{\tanh}=\alpha \;\tanh\left(\omega g\right)$ induces adversarial directions with systematically lower Euclidean energy than the classical FGSM perturbation $\delta_{\mathrm{FGSM}}=\varepsilon \;\mathrm{sign}\left(g\right)$, under mild assumptions on the gradient field. Specifically, the resulting perturbations satisfy

(13) $$∥\delta_{\tanh}{∥}_{2}<{∥\delta_{\mathrm{FGSM}}∥}_{2},$$

indicating that gradient saturation effectively suppresses excessive high-frequency components in the perturbation.

This property is central to understanding how smoothing alters the geometry of adversarial perturbations. By reducing the $L_{2}$ energy while preserving directional alignment with the gradient, the smoothed operator produces perturbations that are spatially more coherent and less fragmented. This geometric behavior is consistent with the empirical observations reported in Section 3.2, Section 3.3 and Section 3.4 of the main text.

### 3.6. The Integrated SD-SGSM Framework

Having established the individual components, we synthesize them into a unified adversarial attack framework, termed SD-SGSM. This framework is designed to orchestrate a “surgical strike” on the target model: it maximizes damage to critical semantic features while minimizing collateral perceptual damage to the global image structure. **Synergistic Mechanism.** The core innovation of SD-SGSM lies in the dynamic coupling between domain identification and gradient smoothing. Unlike standard FGSM which blindly applies a uniform ϵ across the entire image lattice I, our framework modulates the perturbation magnitude $\epsilon_{i,j}$ based on the spatial sensitivity map derived in Section 3.2. Formally, we define a pixel-wise cost map $E\in R^{H\times W}$:

$$E_{i,j}=I\left(\left(i,j\right)\in \Omega^{★}\right)\cdot \epsilon_{\mathrm{strong}}+I\left(\left(i,j\right)\notin \Omega^{★}\right)\cdot \epsilon_{\mathrm{weak}},$$

where I(·) is the indicator function and $\Omega^{★}$ is the identified decision-dependent domain. This map E serves as the constraint boundary for the tanh-smoothed update rule defined in Section 3.4.

**Optimization Trade-off.** This spatially adaptive allocation achieves a dual objective: Maximizing Attack Success Rate (ASR): By permitting a larger cost $\epsilon_{\mathrm{strong}}$ exclusively within the decision-dependent domain $\Omega^{★}$, the algorithm can overcome local gradient masking and effectively disrupt the features most relied upon by the classifier. Minimizing Global Distortion ($\ell_{p}$ Norm): By strictly constraining the vast majority of the image (the non-sensitive background $\bar{R}$) to a lower cost $\epsilon_{\mathrm{weak}}$, we significantly reduce the overall perturbation energy.

Consequently, SD-SGSM breaks the conventional dependency between high ASR and high distortion. It demonstrates that by focusing the perturbation cost where it matters most, one can achieve superior evasion performance with a reduced global $\ell_{2}$ or $\ell_{\infty }$ footprint. The complete procedure is summarized in Algorithm 1.

To implement the proposed Spatially Distributed Perturbation Strategy (SDPS), we design a unified framework that dynamically adapts the attack strength to the model’s regional sensitivity. The complete procedure is outlined in Algorithm 1.

**Decision-Dependent Domain Identification.** The core premise of SDPS is that not all image regions are equally robust. We first employ a scanning mechanism to locate the decision-dependent domain $\Omega^{★}$. By systematically applying the local zero-out operator $Z_{\Omega }$ (as defined in Equation (1)) and monitoring the classifier’s confidence on the ground truth label *y*, we identify the region where evidence removal causes the most significant drop in confidence ($\gamma_{\min}$). This region $\Omega^{★}$ represents the spatial support most critical to the model’s current prediction.
**Algorithm 1:** Spatially Distributed Perturbation Strategy with SGSM (SD-SGSM)
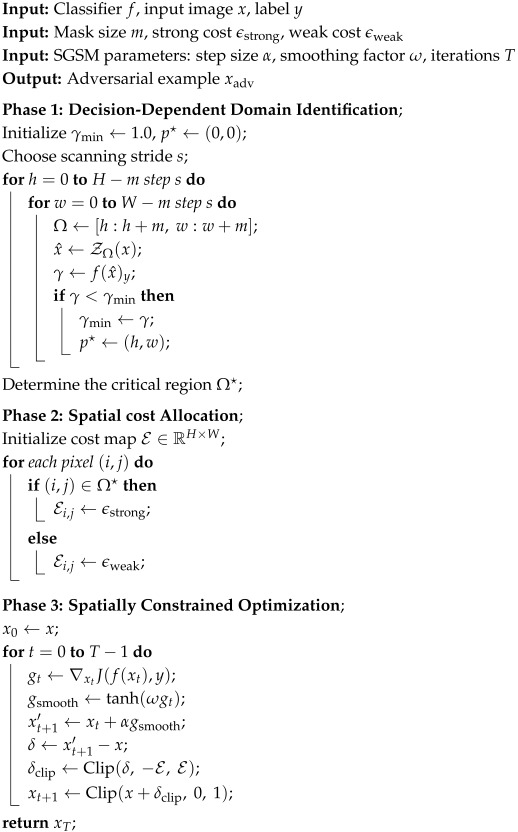


**Spatial Cost Allocation.** Unlike standard FGSM variants that enforce a uniform $\ell_{\infty }$ constraint (a single scalar ϵ for the whole image), we construct a spatial cost map $E\in R^{H\times W}$. Guided by the domain identification in Phase 1, we allocate a larger perturbation cost $\epsilon_{\mathrm{strong}}$ specifically to the decision-dependent domain $\Omega^{★}$ to maximize feature disruption. Conversely, the background region is restricted to a strictly lower cost $\epsilon_{\mathrm{weak}}$. The mechanism prioritizes allocating greater perturbation to regions of high model uncertainty, while actively suppressing noise in confident areas to limit the overall image-wide perturbation.

**Spatially Constrained Optimization.** In this phase, we generate the adversarial example by integrating the Smoothed Gradient Sign Method (SGSM) with our spatial constraints.

**Gradient Smoothing.** Instead of using the standard sign(·) function—which causes gradient quantization and oscillation around decision boundaries—we employ a hyperbolic tangent transformation: $g_{\mathrm{smooth}}=\tanh\left(\omega \cdot g_{t}\right)$. This operation smooths the gradient steps, providing continuous magnitude adjustments that allow for more precise navigation of the loss landscape compared to the binary updates of I-FGSM.

**Spatial Projection.** The synergy of our method culminates in the projection step. The perturbation δ generated by SGSM is clipped element-wise against the spatial cost map E constructed in Phase 2 ($|\delta_{i,j}|\leq E_{i,j}$). This ensures that the high-intensity, smooth perturbations are strictly confined to the critical domain $\Omega^{★}$, effectively realizing our dual objective: maximizing the attack success rate via optimized gradients while minimizing the global $\ell_{2}$ norm via spatial masking.

### 3.7. The Multi-Step SD-SGSM Framework

To ensure a comprehensive and objective assessment against state-of-the-art white-box attacks—most of which employ iterative optimization (e.g., TI-FGSM, APGD, FAB)—we formulate SD-SGSM as a flexible framework adaptable to both single-step and multi-step configurations. While the single-step variant offers a direct comparison to FGSM, we generalize the method to a multi-step iterative setting by incorporating repeated gradient updates with explicit spatial projections at each step. This allows us to rigorously evaluate the proposed method against established iterative baselines under a unified threat model.

**Iterative Update.** At each iteration t=0,…,T−1, let $x_{t}$ denote the current adversarial example. The unconstrained update via the Smoothed Gradient Sign Method (SGSM) is

(14) $$x_{t+1}^{\prime }=x_{t}+\alpha \;\tanh\left(\omega \;\nabla_{x_{t}}J\left(f\left(x_{t}\right),y\right)\right),$$

where $x_{t+1}^{\prime }$ is a temp variable for adversarial examples, indicating an intermediate value during the computation, the perturbation relative to the original input *x* is

(15) $$\delta_{t}=x_{t+1}^{\prime }-x.$$

**Spatial Projection.** To enforce the pixel-wise cost map E, the perturbation is clipped element-wise:

(16) $$\delta_{t}^{\mathrm{clip}}\left(i,j\right)=\min\left(\max\left(\delta_{t}\left(i,j\right),-E_{i,j}\right),E_{i,j}\right),\;\forall \left(i,j\right),$$

ensuring $|\delta_{t}\left(i,j\right)|\leq E_{i,j}$ at every iteration.

**Validity Projection.** The adversarial example is then projected onto the valid input domain:

(17) $$x_{t+1}=\mathrm{Clip}\left(x+\delta_{t}^{\mathrm{clip}},0,1\right).$$

**Per-Iteration Constraint.** Combining the above, the multi-step update satisfies

(18) $$|\delta_{t}\left(i,j\right)|\leq E_{i,j},\;\forall t=0,\ldots ,T-1,\;\forall \left(i,j\right)\in I,$$

which guarantees localized perturbations while controlling global distortion. This formulation preserves the localized aggression principle of SD-SGSM, allowing stronger perturbations in critical regions while maintaining low overall perceptual impact.

## 4. Experiments

### 4.1. Datasets

In the context of classification tasks, we employ the validation images from the Mini-ImageNet, CIFAR-10 [29], datasets for our experiments. As target classifiers, we utilize pretrained ResNet18 models.

Mini-ImageNet: The Mini-ImageNet, a subset extracted from the expansive ImageNet dataset, maintains a moderately scaled collection of data, playing a pivotal role in academic research within the realm of image. Comprising 100 distinct categories, it encompasses a wide array of object types, including various animals, plants, daily necessities, and more. Each category, on average, contains approximately 600 image examples, ensuring a robust and diverse representation. The richness and diversity of the image content closely align with the real-world appearance of these objects. The dataset includes images captured under diverse angles, lighting conditions, and other situational variables, effectively challenging classification models’ abilities to navigate complex visual scenarios.

CIFAR-10 [29]: The CIFAR-10 dataset consists of 60,000 color images, each possessing a resolution of 32 × 32 pixels, evenly distributed among 10 distinct classes. The dataset is divided into two subsets: 50,000 images allocated for training and 10,000 images set aside for testing, facilitating the assessment of model performance.

### 4.2. Evaluation Metrics

To enable a rigorous and comprehensive assessment of the proposed SD-SGSM, several evaluation metrics are employed. These metrics jointly characterize the model’s robustness, perturbation properties, perceptual quality, and computational overhead.

**Clean Accuracy (Clean Acc, %).** The clean accuracy reflects the classifier’s baseline performance on unperturbed test samples. A high clean accuracy ensures that subsequent performance changes can be attributed to adversarial perturbations rather than model deficiencies.

**Attack Success Rate (ASR, %).** The attack success rate quantifies the proportion of adversarial examples that successfully induce misclassification. It is defined as:

$$\mathrm{ASR}=\frac{n}{N}\times 100\%$$

where *N* denotes the total number of evaluated samples and *n* denotes the number of samples for which the predicted label is altered. This metric directly reflects the effectiveness of the attack.

**Perturbation Strength (using $L_{\infty }$ and $L_{2}$ norms).** The magnitude of adversarial perturbations is quantified using the $L_{\infty }$ and $L_{2}$ norms. Reporting the mean and standard deviation, or the median, captures both the central tendency and the variance of perturbation intensity. The $L_{\infty }$ norm constrains the maximum per-pixel deviation, whereas the $L_{2}$ norm measures the overall perturbation energy.

**Perceptual Similarity (SSIM).** The structural similarity index (SSIM) is employed to evaluate the perceptual closeness between clean images and their adversarial counterparts. Higher SSIM values indicate stronger perceptual consistency, which is essential for realistic and stealthy adversarial examples.

**Computational Efficiency (Time per Sample).** The average time required to generate a single adversarial example is recorded to quantify computational efficiency. This metric is crucial for evaluating the practicality of an attack, particularly in large-scale or real-time scenarios.

### 4.3. Experimental Setup

**Implementation Details.** All experiments were conducted on a computational platform equipped with an NVIDIA GeForce RTX 4070 Laptop GPU (8 GB VRAM). The proposed method and all baselines were implemented using the PyTorch framework (version 2.5.1).

**Fairness Assurance and Parameter Configuration.** To ensure a rigorous and fair comparison, we strictly unified the perturbation constraints and hyperparameter settings across all evaluated methods. We benchmarked SD-SGSM against a comprehensive suite of white-box attacks, including single-step methods (e.g., FGSM) and multi-step FGSM variants (e.g., MI-FGSM, NI-FGSM, TI-FGSM), as well as other state-of-the-art algorithms (e.g., PGD, APGD).

Standardization was strictly enforced as follows:

- **Perturbation Budget ($L_{\infty }$ Constraint):** All baseline methods were evaluated under a uniform $L_{\infty }$ norm constraint with a maximum perturbation budget of ϵ=8/255 applied to every pixel across the entire image.
- **Step Size (α):** For all gradient-based iterative methods (including PGD, APGD, and the multi-step version of SD-SGSM), the step size was unified at α=4/255 to maintain consistency in the optimization trajectory.
- **Constraint on SD-SGSM:** Regarding the spatially heterogeneous strategy, we explicitly clarify that our method operates strictly within the standard threat model. While SD-SGSM dynamically allocates perturbation budgets based on regional importance, the perturbation magnitude in both decision-critical regions and background regions is strictly bounded by the global limit ϵ=8/255. In other words, the perturbation at any pixel location never exceeds the maximum budget allowed for baseline methods.

## 5. Experimental Results

### 5.1. Ablation Study

To dissect the functional role of each component within the proposed Spatially Distributed Smoothed Gradient Sign Method (SD-SGSM), an ablation study was conducted with a pre-trained ResNet18 under four distinct configurations: vanilla FGSM, Smoothed Gradient Sign Method (SGSM), Spatially Distributed FGSM (SD-FGSM), and the complete SD-SGSM framework. We evaluated these variants on the CIFAR-10 dataset under an $L_{\infty }$ constraint of ϵ=8/255 (Table 1).

The baseline FGSM yielded an Attack Success Rate (ASR) of 82.6%. When the sign function was replaced with the tanh function (SGSM), the perturbation magnitude was significantly reduced, with the $L_{2}$ norm dropping from 1.724 [±0.040] to 1.432 [±0.179]. Although this smoothing slightly decreased the ASR to 82.1%, it notably improved the perceptual quality, increasing the Structural Similarity Index (SSIM) to 0.9427 [±0.0393]. Conversely, the introduction of spatial distribution (SD-FGSM) enhanced the attack strength, raising the ASR to 83.6%, but at the cost of higher distortion levels. The proposed SD-SGSM combined the benefits of both strategies, achieving a robust ASR of 83.2% while maintaining a low $L_{2}$ norm of 1.466 [±0.175] and a high SSIM of 0.9410 [±0.0400]. Despite a moderate increase in computational overhead (0.08 s per sample), the SD-SGSM provided the most favorable trade-off between attack success and image fidelity. Together, these data show that spatial distribution and gradient smoothing are complementary mechanisms that enhance attack effectiveness while preserving visual quality.

### 5.2. Performance Comparison Between SD-SGSM and FGSM Variants

We subsequently benchmarked SD-SGSM against established momentum-based iterative attacks, including MI-FGSM [30], NI-FGSM [31], and TI-FGSM [32]. All methods were evaluated under a fixed perturbation cost (ϵ=8/255). It is crucial to note that although SD-SGSM employs a spatially distributed strategy, the perturbation magnitude at any individual pixel is strictly bounded by this global ϵ.

**Figure 3:**
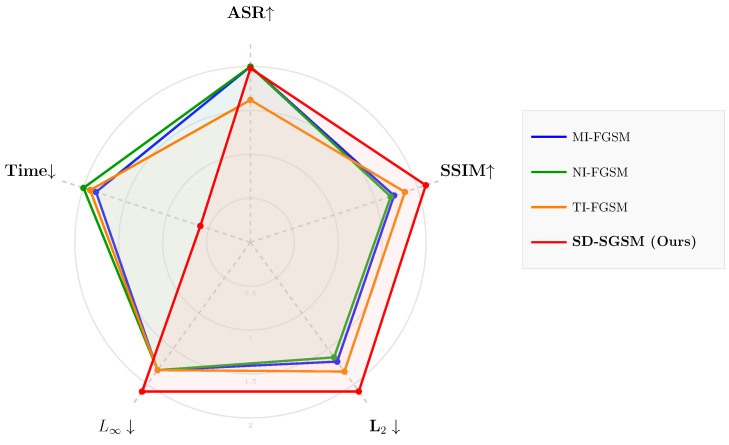
Radar chart comparison of adversarial attacks. Metrics are normalized such that larger areas represent better overall performance (higher ASR/SSIM, lower $L_{2}$/$L_{\infty }$/Time). The arrows indicate the direction of better performance.

**Figure 4:**
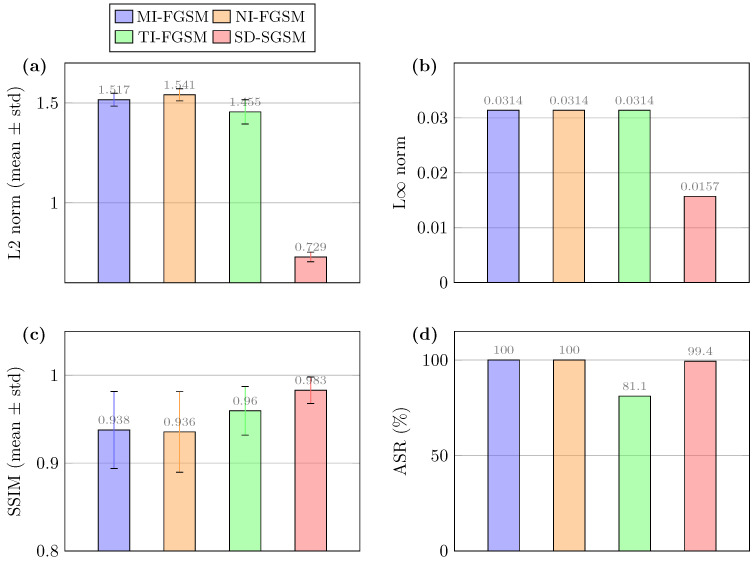
Quantitative comparison of attack methods across (**a**) $L_{2}$ norm, (**b**) $L_{\infty }$ norm, (**c**) SSIM, and (**d**) ASR and runtime. SD-SGSM balances high success rates with low distortion.

To ensure a fair and rigorous comparison, all experiments were conducted under a strictly unified parameter configuration. Given that iterative algorithms generally yield finer, more imperceptible perturbations and represent the mainstream evolution of FGSM-based variants, we focused on the multi-step evaluation setting. Specifically, since the established baselines (MI-FGSM, NI-FGSM, and TI-FGSM) operate as iterative white-box attacks, we implemented the multi-step version of our proposed SD-SGSM. Accordingly, the step size α was set to 4/255 and the number of iterations was uniformly set to 10 for all methods to ensure computational consistency. The radar chart (Figure 3) and detailed metric comparisons (Figure 4) illustrate the performance landscape across normalized metrics.

SD-SGSM achieved a near-perfect ASR of 99.4%, which was comparable to and not significantly different from MI-FGSM and NI-FGSM (100.0%). However, in terms of image quality, SD-SGSM significantly outperformed the competing methods. The proposed method yielded the lowest mean $L_{2}$ distortion of 0.729 [±0.024], compared to 1.517 [±0.032] for MI-FGSM and 1.541 [±0.030] for NI-FGSM. Furthermore, SD-SGSM maintained a high SSIM of 0.983 [±0.015], exceeding the perceptual quality of both MI-FGSM and NI-FGSM. Although the per-sample inference time for SD-SGSM was higher (0.181 s) due to the additional computational steps, the method successfully minimized adversarial noise. SD-SGSM delivers competitive attack rates while significantly mitigating perceptual degradation compared to standard iterative baselines.

### 5.3. Comparison with State-of-the-Art White-Box Attacks

To comprehensively evaluate the performance of SD-SGSM, we conducted comparative experiments against eight representative state-of-the-art (SOTA) white-box attack algorithms on the CIFAR-10 dataset. The target model is the ResNet-18 trained in Section 5.2, which achieves a clean accuracy of 96%. The baseline methods include:

- **Gradient-based methods:** PGD [33], Jitter [34];
- **Optimization-based methods:** CW ($L_{2}$) [35], DeepFool [36], FAB [37];
- **Auto-parameter methods:** APGD (CE & DLR loss) and AutoAttack [38].

To ensure a fair comparison, we standardized the hyperparameters across all iterative algorithms. Specifically, the maximum number of iterations was set to T=10, and the maximum perturbation budget was restricted to ϵ=8/255. For gradient-based iterative methods (e.g., PGD, APGD), the step size was unified at α=4/255. For SD-SGSM, we employed the dual perturbation setting ($\epsilon_{g}=4/255,\epsilon_{l}=4/255$) as analyzed in previous sections.

The quantitative comparison results are presented in Table 2, where the best results are highlighted in bold. Since most baseline methods are iterative white-box attacks operating in a high-dimensional gradient space, they generally achieve near-saturated success rates. As observed, PGD, APGD, and AutoAttack reached 100% ASR. Our SD-SGSM achieved 99.40%, which is comparable to the top-performing baselines, demonstrating its robustness in white-box settings. In terms of image fidelity, optimization-based methods like FAB and DeepFool seek the minimal perturbation boundary, thus yielding the lowest $L_{2}$ norms (0.181 and 0.205) and highest SSIM scores. However, compared to standard iterative attacks like PGD ($L_{2}=1.280$) and APGD ($L_{2}=1.270$), SD-SGSM demonstrates significantly superior visual quality. It reduces the $L_{2}$ norm to 0.729 (a ∼43% reduction compared to PGD) and improves SSIM to 0.983, regarding computational efficiency, CW requires the least inference time (0.051 s). However, its ASR (99.30%) is slightly lower than that of SD-SGSM (99.40%). Conversely, while FAB achieves the best perceptual metrics, its inference latency (0.717 s) is nearly four times that of SD-SGSM, making it less practical for real-time applications. SD-SGSM strikes an optimal balance between attack effectiveness, visual quality, and computational efficiency. Even when compared to domain-leading white-box algorithms, SD-SGSM maintains a competitive edge, particularly in generating high-quality adversarial examples with low computational overhead.

### 5.4. Adversarial Perturbations Target Semantically Sensitive Regions

**Figure 5:**
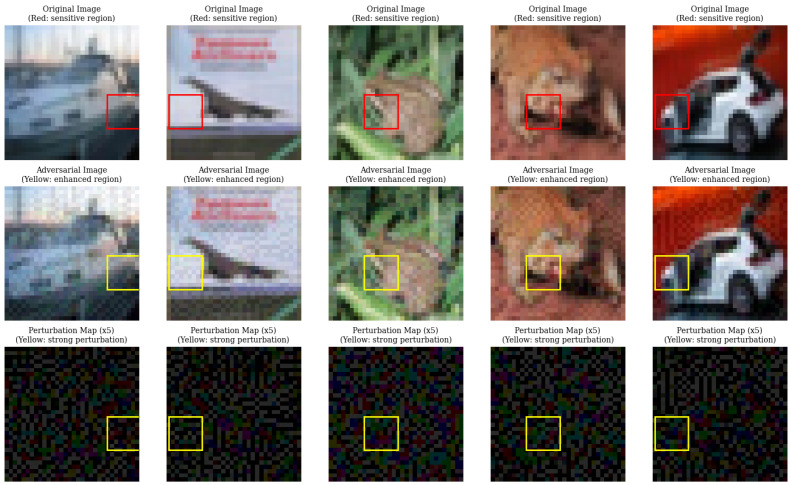
Visualization of SD-SGSM attacks on CIFAR-10. The alignment between sensitive regions (red) and high-perturbation areas (yellow) indicates targeted noise injection.

**Figure 6:**
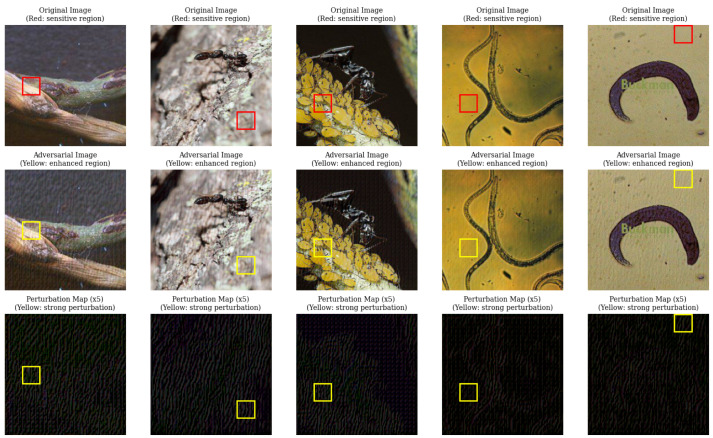
Visualization of SD-SGSM attacks on Mini-ImageNet. The third row displays the 5× amplified perturbation map, showing structural correspondence with the critical object features.

To investigate the mechanism underlying the efficacy of SD-SGSM, we visualized the generated adversarial examples and their corresponding perturbation maps on CIFAR-10 (Figure 5) and Mini-ImageNet (Figure 6) using a ResNet-18 model. We identified “sensitive regions” in the original images (indicated by red bounding boxes) where occlusion induced the sharpest drop in classification confidence.

Qualitative analysis revealed a strong spatial alignment between these model-sensitive areas and the regions of highest perturbation intensity generated by SD-SGSM (indicated by yellow and red bounding boxes). Unlike uniform noise distributions, the perturbation maps amplified by a factor of 5 showed that SD-SGSM concentrates the adversarial cost on discriminative features. For instance, in the Mini-ImageNet samples, the high-scoring tiles in the perturbation map consistently overlapped with the object foreground identified as critical by the occlusion test. The algorithm does not only rely on random noise but rather learns to target the semantic vulnerabilities of the CNN.

## 6. Conclusions

In this work, we introduced the Spatially Distributed Smoothed Gradient Sign Method (SD-SGSM), an adversarial attack framework capable of operating in both single-step and multi-step iterative configurations. By synergistically combining gradient smoothing with region-specific perturbation allocation, the proposed method adapts to varying computational constraints. Our extensive experimental evaluation on the CIFAR-10 dataset validates this flexibility: in the single-step setting, SD-SGSM outperforms the baseline FGSM, while in the multi-step setting, it achieves competitive performance against state-of-the-art iterative white-box algorithms. SD-SGSM achieves a superior trade-off between attack efficacy, perceptual fidelity, and computational efficiency.

Specifically, ablation studies reveal that the tanh-based gradient smoothing (SGSM) effectively reduces perturbation magnitude while maintaining high attack success, improving perceptual similarity as measured by SSIM. Spatially distributed perturbation costs further enhance the adversarial strength, allowing SD-SGSM to concentrate perturbations in decision-critical regions, thereby achieving higher Attack Success Rate (ASR) without introducing excessive distortion. The combination of these components yields the most favorable distortion–success balance, outperforming both conventional FGSM variants and the intermediate SD-FGSM configuration.

Comparative evaluation against momentum-based FGSM attacks (MI-FGSM, NI-FGSM, TI-FGSM) confirms that SD-SGSM consistently delivers lower $L_{2}$ distortion, competitive ASR, and improved structural similarity, while maintaining a moderate computational overhead. The radar and detailed metric analyses collectively highlight the method’s effectiveness across multiple performance dimensions, illustrating that spatial allocation and gradient smoothing operate in a complementary manner.

SD-SGSM offers a practical and computationally efficient alternative to conventional multi-step iterative attacks, enabling high attack effectiveness while maintaining minimal perceptual disturbance at the system input level. Beyond its immediate performance gains, the proposed approach illustrates how incorporating spatial awareness and gradient regularization can reveal structured vulnerabilities inherent to deep learning-based perceptual subsystems. From a systems perspective, these findings suggest that adversarial robustness should be assessed not only through global perturbation costs but also through spatially heterogeneous sensitivity patterns that govern decision-critical information flows. Future work will investigate adaptive spatial allocation mechanisms and evaluate the transferability of the proposed framework across larger-scale datasets, diverse network architectures, and more complex AI-enabled systems.

## Figures and Tables

**Table 1:** Ablation study of SD-SGSM under ϵ=8/255 using a pre-trained ResNet18 model (96% accuracy). Metrics are reported as mean [± std]. Arrows (↑/↓) indicate performance relative to the FGSM baseline.

Method	ASR (%)	$L_{2}$ Norm	$L_{\infty }$ Norm	SSIM	Steps	Time (s)
FGSM	82.6	1.724 [±0.040]	0.0314	0.9225 [±0.052]	1	0.010
SGSM	82.1 ↓	**1.432 [±0.179]**↓	0.0314	**0.9427 [±0.039]**↑	1	0.011
SD-FGSM	83.6 ↑	1.752 [±0.041] ↑	0.0549	0.9210 [±0.053] ↓	1	0.083
SD-SGSM	**83.2**↑	**1.466 [±0.175]**↓	**0.0549**	**0.9410 [±0.040]**↑	1	0.081

**Table 2:** Comparison of SD-SGSM with state-of-the-art white-box attacks (1000 samples). ϵ denotes the $L_{\infty }$ constraint.

Method	Steps	ϵ	ASR (%)	$L_{2}$ Norm	$L_{\infty }$ Norm	SSIM	Time (s)
PGD	10	8/255	**100.00**	1.280 ± 0.047	0.0314	0.955 ± 0.033	0.094
JITTER	10	8/255	92.40	1.161 ± 0.047	0.0314	0.961 ± 0.030	0.117
CW	10	-	99.30	0.355 ± 0.158	0.0100 ± 0.006	0.996 ± 0.004	**0.051**
DEEPFOOL	10	-	99.60	0.205 ± 0.163	0.0248 ± 0.019	0.998 ± 0.003	0.194
FAB	10	8/255	99.90	**0.181 ± 0.129**	**0.0041 ± 0.003**	**0.999 ± 0.002**	0.717
APGD	10	8/255	**100.00**	1.270 ± 0.046	0.0314	0.956 ± 0.033	0.122
APGD-DLR	10	8/255	**100.00**	1.296 ± 0.198	0.0314	0.951 ± 0.039	0.140
AUTOATTACK	-	8/255	**100.00**	1.274 ± 0.046	0.0314	0.955 ± 0.033	0.131
SD-SGSM (Ours)	10	8/255	99.40	0.729 ± 0.024	0.0157	0.983 ± 0.015	0.181

## Data Availability

The data presented in this study are available in the University of Toronto website at https://www.cs.toronto.edu/~kriz/cifar.html (accessed on 3 January 2026), reference number [30]. These data were derived from the following resources available in the public domain: The CIFAR-10 dataset (https://www.cs.toronto.edu/~kriz/cifar-10-python.tar.gz, accessed on 3 January 2026). The code is publicly available at https://github.com/dawei7777/SDPS-SGSM, accessed on 3 January 2026.

## Appendix A. Proofs of Propositions

### Appendix A.1. Statement and Preliminaries for Proposition 1

Proposition 1 establishes that the perturbation induced by the smoothed operator $\delta_{\tanh}=\alpha \;\tanh\left(\omega g\right)$ exhibits strictly smaller Euclidean energy than the classical FGSM perturbation $\delta_{\mathrm{FGSM}}=\varepsilon \;\mathrm{sign}\left(g\right)$, under mild regularity assumptions on the gradient field. Formally, the target inequality is

(A1) $$∥\delta_{\tanh}{∥}_{2}<{∥\delta_{\mathrm{FGSM}}∥}_{2}.$$

This result is conceptually relevant because it characterizes how gradient saturation modifies the geometry of adversarial perturbations. A reduction in $L_{2}$ energy directly relates to smoother, more coherent perturbation directions, consistent with the behavior discussed in Section 3.2, Section 3.3 and Section 3.4 of the main text.

Our derivation relies on two ingredients:

**Gain-matching condition.** The scaling factor α is defined via

(A2) $$\alpha \;\langle g,\tanh\left(\omega g\right)\rangle ={\varepsilon \;∥g∥}_{1},$$

ensuring that the projected update magnitude along *g* matches that of FGSM.

**Local linearity of the activation.** In the regime where the gradient lies within the linear part of the hyperbolic tangent, we use

(A3) tanh(ωg)≈ωg,

which yields closed-form expressions for the comparison.

Under these assumptions, inequality (A1) follows from the argument detailed next.

### Appendix A.2. Proof of Proposition 1

**Proof.** Recall the statement of Proposition 1: When ω>0 is sufficiently small for $\tanh(\omega g_{i})$ to operate in its linear regime (i.e., $\tanh\left(\omega g_{i}\right)\approx \omega g_{i}$), the Tanh-based perturbation necessarily satisfies

(A4) $$∥\delta_{\tanh}{∥}_{2}^{2}\leq \varepsilon^{2}n={∥\delta_{\mathrm{FGSM}}∥}_{2}^{2}.$$

Starting from the gain-matching condition (A2), we solve for the scaling factor:

(A5) $$\alpha =\varepsilon \;\frac{{∥g∥}_{1}}{\langle g,\tanh(\omega g)\rangle }.$$

By Equation (9), we have

(A6) $$\delta_{\tanh}=\alpha \tanh\left(\omega g\right).$$

Substituting (A5) into the squared $L_{2}$ norm of the smoothed perturbation yields

(A7) $$∥\delta_{\tanh}{∥}_{2}^{2}=\alpha^{2}{∥\tanh\left(\omega g\right)∥}_{2}^{2}=\varepsilon^{2}\frac{{∥g∥}_{1}^{2}}{{\langle g,\tanh\left(\omega g\right)\rangle }^{2}}\;{∥\tanh\left(\omega g\right)∥}_{2}^{2}.$$

Applying the local linearity approximation (A3), we obtain

(A8) $$\begin{array}{cc} \langle g,\tanh(\omega g)\rangle \; & ={\omega ∥g∥}_{2}^{2}, \end{array}$$

(A9) $$\begin{array}{cc} {∥\tanh\left(\omega g\right)∥}_{2}^{2}\; & =\omega^{2}{∥g∥}_{2}^{2}. \end{array}$$

Substituting (A8) and (A9) into (A7) yields

(A10) $$∥\delta_{\tanh}{∥}_{2}^{2}=\varepsilon^{2}\frac{{∥g∥}_{1}^{2}}{{(\omega ∥g∥}_{2}^{2}{)}^{2}}\;(\omega^{2}{∥g∥}_{2}^{2})=\varepsilon^{2}\;\frac{{∥g∥}_{1}^{2}}{{∥g∥}_{2}^{2}}.$$

We now invoke the Cauchy–Schwarz inequality on the vector of absolute gradients |g| and the all-ones vector 1:

(A11) $${∥g∥}_{1}=\sum_{i=1}^{n}|g_{i}|\cdot 1\leq \sqrt{\sum_{i=1}^{n}{\left|g_{i}\right|}^{2}}\cdot \sqrt{\sum_{i=1}^{n}1^{2}}={∥g∥}_{2}\sqrt{n}.$$

Squaring both sides yields ${∥g∥}_{1}^{2}\leq n{∥g∥}_{2}^{2}$, or equivalently,

(A12) $$\frac{{∥g∥}_{1}^{2}}{{∥g∥}_{2}^{2}}\leq n.$$

Therefore, $∥\delta_{\tanh}{∥}_{2}^{2}\leq \varepsilon^{2}n$. By Equation (8), we have

(A13) $$∥\delta_{\mathrm{FGSM}}{∥}_{2}^{2}=\varepsilon^{2}n$$

Thus,

(A14) $$∥\delta_{\tanh}{∥}_{2}^{2}\leq \varepsilon^{2}n={∥\delta_{\mathrm{FGSM}}∥}_{2}^{2}.$$

The equality in the Cauchy–Schwarz inequality holds if and only if $|g_{i}|$ is proportional to 1 for all *i*. In any practical scenario where gradients vary across dimensions, the strict inequality holds:

(A15) $$∥\delta_{\tanh}{∥}_{2}<{∥\delta_{\mathrm{FGSM}}∥}_{2}.$$

□
